# Comparison of sex differences on outcomes after aneurysmal subarachnoid hemorrhage: a propensity score-matched analysis

**DOI:** 10.1186/s12883-024-03659-3

**Published:** 2024-05-04

**Authors:** Yuwei Han, Bingying Zhang, Xin Qi, Guanqian Yuan, Xiaoming Li, Guangzhi Hao, Guobiao Liang

**Affiliations:** Department of Neurology, General Hospital of Northern Theater Command, NO.83, Wenhua Road, Shenhe District, Shenyang, 110016 Liaoning China

**Keywords:** Aneurysmal subarachnoid hemorrhage, Female, Propensity score matching, Outcome

## Abstract

**Objective:**

Sex differences in outcomes of patients with aneurysmal subarachnoid hemorrhage (aSAH) remain controversial. Therefore, the aim of this study was to investigate the sex differences in the prognosis of patients with aSAH.

**Methods:**

This study retrospectively analyzed the clinical data of aSAH patients admitted to the Department of Neurosurgery of General Hospital of Northern Theater Command, from April 2020 to January 2022. The modified Rankin Scale (mRS) was used to evaluate outcomes at 3-month post-discharge. Baseline characteristics, in-hospital complications and outcomes were compared after 1:1 propensity score matching (PSM).

**Results:**

A total of 665 patients were included and the majority (63.8%) were female. Female patients were significantly older than male patients (59.3 ± 10.9 years vs. 55.1 ± 10.9 years, *P <* 0.001). After PSM, 141 male and 141 female patients were compared. Comparing postoperative complications and mRS scores, the incidence of delayed cerebral ischemia (DCI) and hydrocephalus and mRS ≥ 2 at 3-month were significantly higher in female patients than in male patients. After adjustment, the analysis of risk factors for unfavorable prognosis at 3-month showed that age, sex, smoking, high Hunt Hess grade, high mFisher score, DCI, and hydrocephalus were independent risk factors.

**Conclusion:**

Female patients with aSAH have a worse prognosis than male patients, and this difference may be because females are more vulnerable to DCI and hydrocephalus.

## Introduction

Aneurysmal subarachnoid hemorrhage (aSAH) is more prevalent in women than in men compared with other types of stroke [1, 2]. Several studies have shown that females are associated with a poorer prognosis after aSAH compared to males [3–5]. Others have reported that sex is not a risk factor for aSAH prognosis [6–8]. Thus, it remains controversial whether outcomes following aSAH differ between men and women.

Aneurysmal characteristics and complications including delayed cerebral ischemia (DCI) and hydrocephalus are the most important factors responsible for the poor prognosis of aSAH [9, 10]. Several studies have shown that these factors may contribute to the sex differences in poor prognosis of aSAH. Other studies have reported sex differences in the site and location of ruptured aneurysms [8, 11].

Therefore, this study retrospectively analyzed the clinical data of aSAH patients treated at our institution. The aim of this study was to investigate sex differences in the aneurysm characteristics, complications and 3-month prognosis.

## Method

### Patients’ material collection and variables

This was a retrospective study, and all protocols were approved by the Ethics Committee of the General Hospital of Northern Theater Command, which was approved on 10 March 2023. Patients diagnosed with aSAH at the Neurosurgery Department of the General Hospital of Northern Theater Command from April 2020 to January 2022 were collected. All data analyses were performed in accordance with the Declaration of Helsinki and local ethical policies. And all patients were treated and managed according to guidelines.

All patients were diagnosed with aSAH by computed tomography angiography (CTA) or digital subtraction angiography (DSA), and the location and number of aneurysms were clarified. If the patient had multiple intracranial aneurysms, the ruptured aneurysm was recorded as the responsible aneurysm. In this study, we have reviewed in detail information on the basic characteristics of patients, smoking and drinking, aneurysm characteristics, treatment approaches, and comorbidities. Aneurysms originating from various segments of the vertebral and basilar arteries and their branching aneurysms were recorded as posterior circulation artery (PCA) aneurysms. Aneurysms originating from the internal carotid artery, anterior and middle cerebral and their branching aneurysms were anterior circulation artery (ACA) aneurysms.In this study, the treatment of all patients was categorized into intervention and craniotomy. Intervention mainly consisted of coil embolization and flow direction. Patients who underwent clipping or bypass were categorized as craniotomies.

We set inclusion criteria including (1) age ≥ 18 years; (2) no history of ruptured aneurysm; (3) patients who underwent only craniotomy and intervention therapy; and (4) time from rupture to admission not exceeding 72 h, and time from admission to treatment not exceeding 72 h. To ensure the accuracy of the analysis, we excluded: (1) other neurological diseases (tumors, vascular malformations, Parkinson’s disease) and functional or neurological deficits of the extremities from any cause; (2) history of neurosurgery prior to the rupture; (3) treatment with external ventricular drainage, lumbar puncture, angiography, intubation and/or mechanical ventilation in other hospitals prior to the visit to our clinic; (4) anemia on admission (hemoglobin < 120 g/L for male and < 110 g/L for female); (5) receiving or have received hormonal replacement therapy and (6) missing clinical data.

### Management and assessment

All patients diagnosed with aSAH were treated with the antivasospastic agent nimodipine after admission. World Federation of Neurosurgical Societies (WFNS) grade, Hunt-Hess grade, and modified Fisher (mFisher) scores were obtained based on the patients’ neurologic function tests and imaging at the time of admission. The presence of cerebral ischemia and hydrocephalus was assessed using the patient’s pre-discharge cranial CT imaging and clinical presentation. We evaluated clinical status using the modified Rankin Scale (mRS) [5, 12] at 3-month after discharge (Neurosurgeons followed up with patients via telephone or an outpatient appointment). Poor functional outcome was defined as mRS ≥ 2. Dead patients were also categorized as having mRS ≥ 2.

### Statistical analysis

Statistical analyses were performed using SPSS version 27 (IBM, Armonk, New York, USA). Values of *P* < 0.05 were considered statistically significant. Continuous variables were described as mean ± standard deviation (SD) and median with interquar-tile range (IQR), and count variables as incidence (percentage). Continuous variables were tested by Student’s t test and Mann-Whitney test. All risk factors with *P* < 0.05 in the univariate analysis were included in the multifactorial logistic regression analysis to identify independent risk factors associated with unfavorable 3-month outcomes. Associations were expressed as odds ratios (OR) and 95% confidence intervals (CI).

## Results

### Patient characteristics

A total of 682 patients diagnosed with aSAH were reviewed in this study. Five patients aged < 18 years were excluded, nine patients with receiving only conservative treatment were excluded, and three patients with missing clinical data were excluded. A predominantly female population (63.8%) of 665 patients with a mean age of 57.8 ± 11.1 years (range, 22–92 years) was included in this study. And the mean age of female patients was significantly higher than that of males (Table 1). More than half of the patients had comorbid hypertension. Approximately one-third of the patients were smokers and one-fifth were alcohol drinkers, a large proportion of whom were male. Aneurysms originated mainly in the anterior circulation artery (92.0%). 63% of the patients underwent intervention and 37% underwent craniotomy treatment. Patients had predominantly low World Federation of Neurosurgical Societies (WFNS) grade, Hunt-Hess grade, and mFisher score. DCI occurred in about a quarter of the patients and hydrocephalus in 11.6%. The mortality rate at discharge was 5.1%, and the incidence of 3-month unfavorable outcome (mRS ≥ 2) was 31.7%. There is no significant difference between the sexes in these variables.

**Table 1:** Characteristics of patients and comparison of sex differences before propensity score matching

Parameter	Total (*n* = 665)	Female (*n* = 424)	Male (*n* = 241)	*P* value
**Age(mean ± SD)**	57.8 ± 11.1	59.3 ± 10.9	55.1 ± 10.9	< 0.001^*^
**Comorbidities**				
Hypertension	361 (54.3%)	232 (54.7%)	129 (53.5%)	0.767
Diabetes	51 (7.7%)	36 (8.5%)	15 (6.2%)	0.291
Smoking	221 (33.2%)	68 (16%)	153 (63.5%)	< 0.001^*^
Drinking	134 (20.2%)	23 (5.4%)	111 (46.1%)	< 0.001^*^
**Aneurysm location**				0.121
PCA	53 (8.0%)	39 (9.2%)	14 (5.8%)	
ACA	612 (92.0%)	385 (90.8%)	227 (94.2%)	
**Maximum diameter of aneurysm** ^**a**^ **(IQR)**	3.85 (3.00–5.00)	3.81 (3.00–5.00)	4.00 (2.70–5.20)	0.874
**Treatment**				0.100
Intervention	419 (63.0%)	277 (65.3%)	142 (58.9%)	
Craniotomy	246 (37%)	147 (34.7%)	99 (41.1%)	
**WFNS**				0.140
I-III	478 (71.9%)	313 (73.8%)	165 (68.5%)	
IV-V	187 (28.1%)	111 (26.2%)	76 (31.5%)	
**Hunt Hess**				0.386
I-III	528 (79.4%)	342 (80.4%)	187 (77.6%)	
IV-V	137 (20.6%)	83 (19.6%)	54 (22.4%)	
**mFisher**				0.489
0–2	489 (73.5%)	308 (72.6%)	181 (75.1%)	
3–4	176 (26.5%)	116 (27.4%)	60 (24.9%)	
**Complications**				
DCI	172 (25.9%)	112 (26.4%)	60 (24.9%)	0.667
Seizures	6 (0.9%)	4 (0.9%)	2 (0.8%)	0.784
Hydrocephalus	77 (11.6%)	49 (11.6%)	28 (11.6%)	0.981
Mortality	34 (5.1%)	21 (5.0%)	13 (5.4%)	0.804
**mRS ≥ 2**	211 (31.7%)	135 (31.8%)	76 (31.5%)	0.935

PCA, posterior circulation artery; ACA, anterior circulation arterial; DCI, delayed cerebral ischemia; mRS, modified Rankin Scale

^a^ Unit of measurement, mm

### Sex differences and adjustment

To adjust for differences in basic patient characteristics such as age, smoking, alcohol consumption, hypertension, diabetes mellitus, aneurysm location and diameter, treatment, WFNS grade, Hunt Hess grade, and mFisher score, we performed 1:1 propensity score matching (PSM) with a matching tolerance of 0.02. After the PSM, we compared the information during hospitalization and prognostic information between males and females.

After PSM, the mean age of the patients was similar in males and females (57.6 ± 10.8 years vs. 57.8 ± 10.8 years, *P* = 0.878). There were no significant differences between males and females in terms of hypertension, diabetes, smoking, drinking, aneurysm location, maximum diameter of aneurysm, treatment approach, WFNS grade, Hunt Hess grade and mFisher (all *P* > 0.05, Table 2). After PSM, female patients had a higher incidence of DCI and hydrocephalus (56/141 [39.7%] vs. 37/141 [26.2%], *P* = 0.016; 28/141 [19.9%] vs. 17/141 [12.1%], *P* = 0.044, Table 2). Furthermore, the number of female patients with mRS score ≥ 2 at 3-month was significantly larger than that of male patients (*P* < 0.001).

**Table 2:** Characteristics of patients and comparison of sex differences after propensity score matching

Parameter	Total (*n* = 282)	Female (*n* = 141)	Male (*n* = 141)	*P* value
**Age(mean ± SD)**	57.7 ± 10.8	57.6 ± 10.8	57.8 ± 10.8	0.878
**Comorbidities**				
Hypertension	138 (48.9%)	65 (46.1%)	73 (51.8%)	0.341
Diabetes	15 (5.3%)	9 (6.4%)	6 (4.3%)	0.426
Smoking	117 (41.5%)	58 (41.1%)	59 (41.8%)	0.904
Drinking	44 (15.6%)	22 (15.6%)	6 (15.6%)	1.000
**Aneurysm location**				0.330
PCA	18 (6.4%)	11 (7.8%)	7 (5.0%)	
ACA	264 (93.6%)	130 (92.2%)	134 (95.0%)	
**Maximum diameter of aneurysm** ^**a**^ **(IQR)**	3.50 (2.95-5.00)	3.50 (3.00-4.88)	3.58 (2.50-5.00)	0.916
**Treatment**				0.809
Intervention	166 (58.9%)	82 (58.2%)	84 (59.6%)	
Craniotomy	116 (41.1%)	59 (41.8%)	57 (40.4%)	
**WFNS**				0.620
I-III	180 (63.8%)	88 (62.4.0%)	92 (65.2%)	
IV-V	102 (36.2%)	53 (37.6%)	49 (34.8%)	
**Hunt Hess**				0.148
I-III	201 (71.3%)	95 (67.4%)	106 (75.2%)	
IV-V	81 (28.7%)	46 (32.6%)	35 (24.8%)	
**mFisher**				0.113
0–2	202 (71.6%)	95 (67.4%)	107 (75.9%)	
3–4	80 (28.4%)	46 (32.6%)	34 (24.1%)	
**Complications**				
DCI	93 (33.0%)	56 (39.7%)	37 (26.2%)	0.016^*^
Seizures	2 (0.7%)	1 (0.7%)	1 (0.7%)	0.999
Hydrocephalus	45 (16%)	28 (19.9%)	17 (12.1%)	0.044^*^
Mortality	24 (8.5%)	14 (9.9%)	10 (7.1%)	0.393
**mRS ≥ 2**	82 (29.1%)	54 (38.3%)	28 (19.9%)	< 0.001^*^

PCA, posterior circulation artery; ACA, anterior circulation arterial; DCI, delayed cerebral ischemia; mRS, modified Rankin Scale

^a^ Unit of measurement, mm

### Risk factors of poor prognosis

The results of univariate and multivariate analyses of 3-month unfavorable outcomes in patients with aSAH are shown in Table 3. Univariate analysis of 665 patients with aSAH showed that age, WFNS grade, Hunt Hess grade, mFisher score, DCI, and hydrocephalus influenced the prognosis of patients at 3-month after discharge. Multivariate analysis logistic regression analysis showed that age (OR = 1.03, 95% CI = 1.005–1.049, *P* = 0.016), high Hunt Hess grade (IV-V) (OR = 4.889, 95% CI = 2.153–11.105, *P* < 0.001), high mFisher score (3–4) (OR = 1.673, 95% CI = 1.006–2.784, *P* = 0.047), DCI (OR = 4.784, 95% CI = 2.977–7.689, *P* < 0.001), and hydrocephalus (OR = 16.322, 95% CI = 7.009–38.009, *P* < 0.001) were independent risk factors for poor prognosis at 3-month.

**Table 3:** Univariate and multivariat analysis of patients with poor prognosis before propensity adjustment

Parameter	Univariate		Multivariate	
	OR (95%CI)	*P* value	OR (95%CI)	*P* value
**Age**	1.028 (1.028–1.044)	< 0.001^*^	1.03 (1.005–1.049)	0.016^*^
**Female**	1.014 (0.722–1.425)	0.935		
**Comorbidities**				
Hypertension	1.085 (0.710–2.367	0.928		
Diabetes	1.562 (0.872–2.799)	0.134		
Smoking	1.363 (0.907–2.441)	0.313		
Drinking	1.078 (0.443–2.044)	0.708		
**Aneurysm location**				
ACA	1.086 (0.853–2.221)	0.505		
**Maximum diameter of aneurysm**	1.276 (1.083–1.620)	0.731		
**Treatment**				
Intervention	1.068 (0.649–1.174)	0.773		
**WFNS**				
IV-V	7.617 (5.229–11.095)	< 0.001^*^	1.551 (0.735–3.276)	0.250
**Hunt Hess**				
IV-V	12.400 (7.943–19.357)	< 0.001^*^	4.889 (2.153–11.105)	< 0.001^*^
**mFisher**				
3–4	4.328 (3.005–6.234)	< 0.001^*^	1.673 (1.006–2.784)	0.047^*^
**Complications**				
DCI	4.613 (3.191–6.669)	< 0.001^*^	4.784 (2.977–7.689)	< 0.001^*^
Hydrocephalus	23.512 (11.441–48.318)	< 0.001^*^	16.322 (7.009–38.009)	< 0.001^*^

ACA, anterior circulation arterial; DCI, delayed cerebral ischemia

After PSM, the results of univariate analysis for 3-month unfavorable outcomes showed that age, sex, smoking, drinking, WFNS grade, Hunt Hess grade, mFisher score, DCI, and hydrocephalus influenced patients’ prognosis at 3 months after discharge (Table 4). Multivariate analysis logistic regression analysis showed that age (OR = 1.068, 95% CI = 1.028–1.110, *P* < 0.001), sex (OR = 5.789, 95% CI = 2.510-13.352, *P* < 0.001), smoking (OR = 1.939, 95% CI = 0.966–3.316, *P* < 0.001), high Hunt Hess grade (IV-V) (OR = 4.980, 95% CI = 1.196–20.737, *P* = 0.027), high mFisher score (3–4) (OR = 3.012, 95% CI = 0.429–21.150, *P* = 0.029), DCI (OR = 4.284, 95% CI = 1.901–9.657, *P* < 0.001), and hydrocephalus (OR = 23.340, 95% CI = 2.859-190.512, *P* < 0.001) were independent risk factors for poor prognosis at 3 months after discharge.

**Table 4:** Univariate and multivariate logistic regression analysis of patients with poor prognosis after propensity adjustment

Parameter	Univariate		Multivariate	
	OR (95%CI)	*P* value	OR (95%CI)	*P* value
**Age**	1.030 (1.007–1.053)	0.011^*^	1.068 (1.028–1.110)	< 0.001^*^
**Female**	3.114 (1.916–5.062)	< 0.001^*^	5.789 (2.510-13.352)	< 0.001^*^
**Comorbidities**				
Hypertension	1.671 (1.073–2.904)	0.096		
Diabetes	1.534 (0.531–4.430)	0.429		
Smoking	1.693 (1.015–3.325)	< 0.001^*^	1.939 (0.966–3.316)	< 0.001^*^
Drinking	1.317 (1.156–2.645)	0.002^*^	1.112 (1.060–2.727)	0.064
**Aneurysm location**				
ACA	1.094 (0.587–1.516)	0.509		
**Maximum diameter of aneurysm**	1.208 (0.993–2.875)	0.110		
**Treatment**				
Intervention	1.067 (1.002–1.641)	0.433		
**WFNS**				
IV-V	9.189 (5.102–16.549)	< 0.001^*^	2.255 (0.637–7.980)	0.207
**Hunt Hess**				
IV-V	13.287 (6.449–27.374)	< 0.001^*^	4.980 (1.196–20.737)	0.027^*^
**mFisher**				
3–4	5.363 (2.955–9.733)	< 0.001^*^	3.012 (0.429–21.150	0.029^*^
**Complications**				
DCI	3.716 (2.178–6.339)	< 0.001^*^	4.284(1.901–9.657)	< 0.001^*^
Hydrocephalus	63.505 (8.604-468.751)	< 0.001^*^	23.340 (2.859-190.512)	0.003^*^

ACA, anterior circulation arterial; DCI, delayed cerebral ischemia

In both sexes, DCI were more common in the anterior circulation than in the posterior circulation. There are gender differences in anterior cerebral artery (ACA) and middle cerebral artery (MCA) (*P =* 0.009, Table 5). The MCA (40%) was the most common site in males, followed by the posterior cerebral artery (PCA) (35%) and ACA (25%). In the female patients, the MCA (58%) was the most common sites of DCI, followed by the PCA (30.4%) and ACA (11.6%). The incidence of poor prognosis was significantly lower in patients with ACA than in patients with MCA. Our results are consistent with those of Adrià Arboix et al., cerebral infarcts in the territory of the anterior cerebral artery have a better prognosis than infarcts in the territory of the middle cerebral artery [13]. In addition, other study has found that MCA infarction has a worse prognosis [14].

**Table 5:** Comparison of prognosis of anterior cerebral artery (ACA) infarction with middle cerebral artery (MCA) and posterior cerebral artery (PCA) infarction in patients with DCI

Parameter	ACA (*n* = 28)	MCA (*n* = 89)	ACA vs. MCA *P* value	PCA (*n* = 55)	ACA vs. PCA *P* value
Sex			0.009^*^		0.181
Female	13 (11.6%)	65 (58.0%)		34 (30.4%)	
Male	15 (25%)	24 (40.0%)		21 (35.0%)	
mRS ≥ 2	6 (21.4%)	40 (44.9%)	0.034^*^	13 (23.6%)	0.821

ACA, anterior cerebral artery; MCA, middle cerebral artery; PCA, posterior cerebral artery; mRS, modified Rankin Scale

### Effect of menopause on prognosis

Age was divided into two groups, ≤ 55 and > 55 years, according to menopause. There were 163 patients ≤ 55 years and 261 patients > 55 years among the 424 female aSAH patients. The incidence of hypertension was significantly higher in female patients > 55 years than in female patients ≤ 55 years (Table 6). Female patients > 55 years had a higher WFNS and mFisher score. In addition, female patients > 55 years had a larger proportion of poor prognosis. Univariate and multivariate logistic analyses of 3-month unfavorable outcomes in female patients with aSAH are shown in Table 7. Univariate analysis of 424 female patients with aSAH showed that > 55 years, WFNS, Hunt Hess, mFisher, DCI and hydrocephalus influenced the prognosis of patients at 3-month after discharge. Based on the results of the univariate analysis evaluating > 55 years, WFNS, Hunt Hess, mFisher, DCI and hydrocephalus, multivariate analysis was performed (Table 7). Multivariate analysis showed that high Hunt Hess grade (IV-V), DCI, and hydrocephalus were independent risk factors for poor prognosis in female aSAH patients.

**Table 6:** Comparison of clinical characteristics in female patients ≤ 55 and > 55 years

Parameter	≤ 55 years (*n* = 163)	> 55 years (*n* = 261)	*P* value
**Comorbidities**			
Hypertension	73 (44.8%)	159 (60.9%)	0.001^*^
Diabetes	11 (6.7%)	25 (9.6%)	0.309
Smoking	25 (15.3%)	43 (16.5%)	0.756
Drinking	11 (6.7%)	12 (4.6%)	0.342
**Aneurysm location**			0.998
PCA	15 (9.2%)	24 (9.2%)	
ACA	148 (90.8%)	237 (90.8%)	
**Maximum diameter of aneurysma (IQR)**	3.80 (3.10–5.20)	3.88 (3.20-5.00)	0.773
**Treatment**			0.602
Intervention	104 (63.8%)	173 (66.3%)	
Craniotomy	59 (36.2%)	88 (33.7%)	
**WFNS**			0.004^*^
I-III	133 (81.6%)	180 (69.0%)	
IV-V	30 (18.4%)	81 (31.0%)	
**Hunt Hess**			0.820
I-III	138 (84.7%)	203 (77.8%)	
IV-V	25 (15.3%)	58 (22.2%)	
**mFisher**			0.005^*^
0–2	131 (80.4%)	177 (67.8%)	
3–4	32 (19.6%)	84 (32.2%)	
**Complications**			
DCI	38 (23.3%)	74 (28.4%)	0.252
Hydrocephalus	14 (8.6%)	35 (13.4%)	0.131
Mortality	7 (4.3%)	14 (5.4%)	0.621
**mRS ≥ 2**	41 (25.2%)	94 (36.0%)	0.020^*^

**Table 7:** Univariate and multivariate analysis of female patients ≤ 55 and > 55 years

Parameter	Univariate		Multivariate	
	OR (95%CI)	*P* value	OR (95%CI)	*P* value
**> 55 years**	1.675 (1.084–2.587)	0.020^*^	1.452 (0.804–2.620)	0.216
**Comorbidities**				
Hypertension	1.051 (0.697–1.585)	0.813		
Diabetes	1.121 (0.815–1.623)	0.056		
Smoking	0.735 (0.411–1.317)	0.301		
Drinking	0.744 (0.287–1.932)	0.544		
**Aneurysm location**				
ACA	0.572 (0.293–1.117)	0.102		
**Maximum diameter of aneurysm**	1.104 (0.788–1.623)	0.711		
**Treatment**				
Intervention	0.901 (0.588–1.380)	0.631		
**WFNS**				
IV-V	7.617 (5.229–11.095)	< 0.001^*^	1.383 (0.525–3.646)	0.512
**Hunt Hess**				
IV-V	11.769 (6.680-20.733)	< 0.001^*^	7.190 (2.529–20.441)	< 0.001^*^
**mFisher**				
3–4	3.896 (2.483–6.114)	< 0.001^*^	1.331 (0.695–2.550)	0.388
**Complications**				
DCI	5.981 (3.746–9.548)	< 0.001^*^	6.586 (3.629–11.956)	< 0.001^*^
Hydrocephalus	22.045 (9.090-53.466)	< 0.001^*^	20.483 (7.205–58.233)	< 0.001^*^

## Discussion

This was a large, single-center, observational retrospective study that involved 665 patients with a clinical diagnosis of aSAH from April 2020 to January 2022. In addition, strict inclusion and exclusion criteria were established to ensure comparability of data and credibility of results. Women (424/665, 63.8%) were more vulnerable to have aSAH compared to men, and women were older. In our study, after adjusting for age, hypertension, diabetes, smoking, drinking, location and size of ruptured aneurysm, WFNS grade, Hunt Hess grade, mFS score, and treatment approach, the results showed that females were more likely to have DCI, with a higher incidence of poor prognosis at 3 months after discharge. In our study, females were approximately twice as likely as males to have a ruptured aneurysm, which is consistent with previous studies [5, 15].

In 665 patients with aSAH, our study found no significant difference in prognosis between male and female [7]. However, the age difference between female and male patients may lead to a comparison bias between the two groups. In addition, the different basic characteristics of aneurysms, comorbidities and treatment approaches in female and male patients may have an influence on the comparison between the two groups [16]. However, in our study, the ratio of female patients with mRS score ≥ 2 was significantly higher than that of male patients after adjusting for age, aneurysm characteristics, comorbidities, WNFS grade, Hunt Hess grade, mFisher score, and treatment approach.

It has been shown that DCI and hydrocephalus are two critical complications that affect the prognosis of aSAH [17]. After PSM, our study found that the incidence of DCI was significantly higher in female aSAH patients than in male patients, consistent with other studies [3, 18–20]. The poor prognosis of females with aSAH may be due to the fact that females are more prone to vasospasm [21]. Studies have shown that early vasospasm predicts a higher incidence of cerebral ischemia associated with subarachnoid hemorrhage and a poorer prognosis [22]. Women have been reported to be an independent risk factor for cerebral vasospasm after aSAH [23, 24]. In addition, catecholamine metabolites reflecting sympathetic excitation have been found to have higher levels in the cerebrospinal fluid of female patients with SAH, which also suggests that they are more prone to vasospasm [25]. Moreover, estrogen may play a protective role in vascular system mainly through its influence on the proliferation and migration of vascular smooth muscle cells [26]. Therefore, because of the decrease of estrogen in postmenopausal women, the role of vascular smooth muscle cells in the vascular system in the physiological and pathological process is affected to a certain extent. This may be a factor in the poor prognosis of some women with ruptured aneurysms, especially those near or past menopause. However, the role and mechanism of estrogen in subarachnoid hemorrhage diseases still need to be further studied.

Furthermore, sharp and abrupt decreases in estradiol concentrations during menopause, combined with genetic predisposition and environmental factors, may disrupt cerebrovascular homeostasis [27]. Increased hemodynamics and oxidative stress may trigger an inflammatory cascade response leading to macrophage, neutrophil, and chemotactic infiltrates that promote chronic inflammation in cerebral arteries. These changes lead to vessel wall degeneration, focal weakening, and cerebral arterial wall ectasia, ultimately leading to aneurysm formation and rupture, thus making postmenopausal women more susceptible to aneurysm rupture leading to aSAH [27].

In addition, after PSM, female patients with aSAH were more vulnerable to hydrocephalus than male patients in our study. Experimental studies in rats have shown that females have a higher risk of hydrocephalus after SAH [28]. In addition, clinical studies have found that females have a high risk of developing hydrocephalus after aSAH [29, 30]. This sex difference may be related to the role of estrogen in maintaining vascular integrity by reducing oxidative stress [27]. In addition, women have higher concentrations of norepinephrine and dopamine metabolites in the cerebrospinal fluid compared to men, therefore women are at a higher risk of developing hydrocephalus [25]. However, reports have shown no finding that female patients with aSAH are more vulnerable to hydrocephalus [20].

Besides confirming that female sex is independent risk factors for poor prognosis after ASAH, our results showed that age, smoking, high Hunt Hess grade, high mFisher score, DCI, and hydrocephalus are independent risk factors of poor prognosis [21]. We observed that high Hunt Hess grade, high mFisher score were more common in women whereas smoking was more frequent in men. In our study, older age was also an independent risk factor for poor prognosis, which is consistent with other studies [31, 32].

We reviewed clinical and imaging records and included only confirmed first-time ruptured intracranial aneurysm cases. Our study found that females were more likely to have aSAH and that females were an independent risk factor for poor prognosis, which may be related to the fact that females are more vulnerable to DCI and hydrocephalus. However, the reasons and mechanisms for this sex difference and whether it is related to estrogen remain unclear [33, 34]. Therefore, a rapid diagnosis and amelioration of DCI and hydrocephalus is important to improve the prognosis of patients with aSAH, especially in women, who have a higher morbidity.

Most studies show that the average size of a ruptured aneurysm is less than 7 mm [35]. Post-rupture thrombosis of the aneurysm lumen may explain why the diameter of many of the ruptured aneurysms in our series was relatively small. An additional factor that has biased our study cohort towards smaller ruptured aneurysms, is the fact that the higher cost of treating larger ruptured aneurysms led to earlier retrieval of care for many of the patients with larger aneurysms. We hope to address this bias in our future studies.

It’s worth mentioning that in our research, the proportion of patients with a history of alcohol consumption was high (134/665, 20.2%), and about 5:1 between men and women with a history of alcohol consumption, which may be related to the habits formed by the cold climate of the region. In this study, in order to exclude the influence of other factors and only consider the effect of gender on the prognosis of aSAH, we matched the propensity scores of hypertensions, diabetes, smoking and drinking in different genders, so we did not investigate the effect of drinking history on the prognosis of SAH. We did not have separate statistics on the effect of alcohol consumption on prognosis. However, it does not mean that alcohol consumption has no effect on aSAH prognosis. Some studies have shown that the patient proportion of alcohol consumption was dramatically greater in males than that in females [36]. Patients with alcohol consumption are at higher odds of angiographic vasospasm and delayed cerebral ischemia, which are related to poor prognosis following aSAH [36]. It remains to be further explored whether alcohol consumption has a significant impact on the prognosis of patients with aSAH by sex. In addition, the relationship between the location of the aneurysm and non-dominant hemispheres is crucial to the prognosis of the patient. Considering that the number of cases in this study is not enough, there are only 141 patients in each group after PSM. If divided according to the location of aneurysms and the non-dominant hemispheres, the number of patients who can be matched after PSM will be very small, which may affect the conclusion. In the next step, we will collect as many cases as possible, further classify the location of the aneurysm, and take into account the important factors such as the location of the aneurysm and the non-dominant hemisphere. In addition, smoking has a dose-dependent and cumulative association with SAH risk [37], whereas smoking was not quantified in our study. Since the number of patients who underwent seizures in this study was very low and the symptoms were transient in duration. It was not possible to analyze the clinical correlation of early seizures in patients with aSAH on clinical prognosis or in-hospital mortality. However, this is a very important issue. Seizures are important complications following aSAH. Seizures are a concern because they may be associated with poor outcomes such as aneurysm rebleeding and additional brain damage, leading to worsened long-term functional outcomes and increased mortality [38]. Therefore, we will supplement the sample size in subsequent studies to keep focusing on the relevance of seizures on the prognosis of patients with aSAH.

As a retrospective study, our study has some limitations. For example, some information related to gender was not collected (e.g., pregnancy and childbirth), which may suggest changes in estrogen levels in female patients and help explore the role of estrogen in female aSAH. In addition, the limited sample size may have prevented us from identifying smaller sex differences. Our study found that females were more likely to have aSAH and that females were an independent risk factor for poor prognosis, which may be related to the fact that females are more vulnerable to DCI and hydrocephalus. However, the reasons and mechanisms for this sex difference and whether it is related to estrogen remain unclear. Since estrogen levels are associated with injury severity and prognosis after aSAH [39], it is necessary to explore the role and mechanism of estrogen in aSAH. We would like to further explore the role and mechanism of estrogen in an animal aSAH model and identify potential therapeutic targets for patients with aSAH.”

## Data Availability

Data on the results of this study are available from the corresponding authors on reasonable request.

## Code availability

Not available.
